# Rationale and protocol paper for the Healthy Active Peaceful Playgrounds for Youth (HAPPY) study

**DOI:** 10.1186/s12889-017-4445-y

**Published:** 2017-05-26

**Authors:** Wayne Cotton, Dean Dudley, Kirsten Jackson, Matthew Winslade, Janice Atkin

**Affiliations:** 1 Sydney School of Education and Social Work, The University of Sydney, Sydney, 2006 Australia; 2 Human Sciences/School of Education, Macquarie University, North Ryde, NSW 2019 Australia; 3 School of Teacher Education, Charles Sturt University, Bathurst, 2795 Australia; 4 Peaceful Playgrounds Australia, Monkerai, 2415 Australia

**Keywords:** Education, Health, Physical activity

## Abstract

**Background:**

A growing body of evidence suggest an association between physical activity levels and students psychological well-being. A number of research studies have evaluated playground interventions that aim to increase physical activity levels, decrease conflict and bullying, and improve students behaviour. The HAPPY Study will evaluate the success of an intervention combining environmental modifications, teacher development, and peer support that can culminate in an easy to implement, low cost and effective model for increasing physical activity, and improving psychological well-being for children.

**Methods/Design:**

Data will be collected at six New South Wales (NSW) primary schools, on physical activity levels, on-task time during classes, and social support for physical activity during a 12 month Cluster Controlled Trial (CT). Three quantitative data collection tools will be used to capture student’s physical activity levels during lunch and recess breaks (the SOPARC tool), student’s on-task behaviour during classes following recess and lunch breaks (the BOSS tool) and where students receive the most encouragement to be physically active from (the Physical Activity Social Support Scale survey). Baseline data will be analysed against follow-up data, collected after an intervention that is rolled out in all schools as part of a stepped wedge CT design.

**Discussion:**

A review of relevant Australian and New Zealand literature suggests that playground interventions can be successful at increasing physical activity levels, increasing social and conflict resolution skills in students, and decreasing incidences of bullying. This study will investigate any correlation between physical activity levels, and student behaviour during classes following breaks.

**Trial Registration:**

Australian and New Zealand Clinical Trials Register ACTRN12616000575437, registered May 2016.

## Background

A multitude of evidence produced from research over the last decade in Australia clearly demonstrates the need for increased physical activity in primary school-aged children [1–3]. There are many health benefits from children being physically active, including greater cardiovascular fitness, lower systolic blood pressure and decreased risk of obesity [1]. Current research suggest that only about half of all Australian primary school aged children are meeting the National Physical Activity recommendations [4] guidelines of 60 min a day of moderate to vigorous physical activity (MVPA) [2].

As well as physical health, being physically active can have behavioural benefits for children, and these benefits to behaviour in primary school students are also becoming increasingly well evidenced [5, 6] and the importance of healthy eating and physical activity has been linked to positive behaviour in classrooms, increased on-task behaviour during class time, and higher levels of social competence [7–11]. The development of many social skills is another psychological benefit children can receive from participating in physical activity during unstructured play time (i.e. recess and lunch breaks. Pellegrini and Blatchford [12] found that social skills such as sharing, negotiation and conflict resolution were fostered through recess and lunchbreak activities.

The Healthy Active Peaceful Playgrounds for Youth (HAPPY) Study, aims to examine the relationship between physical activity and behaviour in primary school students, and to understand the environmental and societal factors that encourage physical activity in primary school students. Physical activity data will be collected before, during, and after the intervention to measure the impact that environmental modifications, social support and teacher training has on physical activity levels. This data collected on students behaviour will be gathered using a survey and direct observations in class time following breaks. Comparison of these data sets should allow for any trends between physical activity, and psychological well-being in students identified and explored.

The HAPPY Study has been designed with consideration to physical activity interventions that have been evaluated, and shown to improve physical activity levels [13, 14] and improve psychological well-being [15]. The HAPPY Study will evaluate how an intervention combining environmental modifications, teacher development, and peer support can culminate in an easy to implement, low cost and effective model for increasing physical activity, and improving psychological well-being for children.

## Methods/Design

### Study Design

The Healthy Active Peaceful Playgrounds for Youth (HAPPY) study is a 12 month, primary school-based intervention and will be evaluated using a cluster controlled trial with a stepped wedge design. Ethics approval has been obtained from an Australian University Human Ethics Committee (HREA5201500676) as well as from the New South Wales Department of Education and Communities (SERAP2015423). The HAPPY Study is registered with the Australian and New Zealand Clinical Trials Registry (ACTRN12616000575437). Principals, teachers and parents will provide written informed consent.

**Fig. 1:**
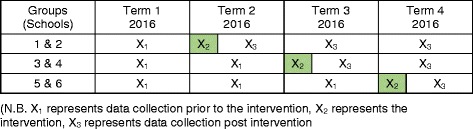
An overview of the study’s stepped wedge design

Following the recruitment process, data will be collected on students’ physical activity levels during lunch and recess, students’ on-task behaviour during class following lunch, and from whom the students receive social support from to be physically active. The intervention will be rolled out in a stepped wedge design. This means that all schools will start as ‘control’ schools and that the intervention will staggered across the 12-month period of the study (three steps in total) with data will being regularly collected across the period (Fig. 1). The stepped wedge design allows for an analysis of the interventions effectiveness and efficacy. As well as having the added benefit of ensuring all schools receive the intervention [16] (i.e., playground markings and teacher training). This benefit is often absent in classic Randomised Control Trials, where control groups may receive no immediate benefit from participating in research studies. This was the main rationale behind adopting this stepped wedge design.

### Sample size calculation

The stepped wedge design used for this study is a variant of a Cluster Controlled Trial where all clusters receive the intervention in a randomised order. Taking into account the possibility of intra-cluster correlation [16], an effect sample size for physical activity observations was calculated using ESS = (m × k/DE) assuming that schools will have an average of 500 students enrolled, the design effect was 0.05. It was calculated that 1661 students would need to be observed to provide adequate power to detect a difference between clusters receiving the intervention.

### Recruitment and study participation

The HAPPY Study will be conducted in six New South Wales Department of Education and Communities (NSWDEC) schools, in the Metropolitan Sydney Region (approx. 33.51°S, 151.12°E). All eligible schools will be sent an invitation to participate by the NSWDEC Schools Sport Unit. Schools that express an interest in participating will be contacted by the research team, and will be provided with more information about the study and intervention.

To participate, schools will be required to provide school level consent for the physical activity observational data collection from the principal, and all students participating in the behavioural, and the social support data collection will be required to provide parent/guardian consent. Teachers will not need to provide consent to participate in the intervention, as no information identifying them will be recorded or reported on.

All six schools will be participate in baseline data collection during the same school term and randomisation into stepped intervention groups will take place after the baseline data.

### Intervention design

The Peaceful Playgrounds Program is a primary school-based package that uses permanent playground markings, teacher training and accompanying resources to promote physical activity in playgrounds, teach fundamental movement skills, and support social skill development in students. The Peaceful Playgrounds Program will be used in the HAPPY Study as the intervention, and the effects of this on both physical activity levels, and behaviour in students measured.

Several research studies using either the Peaceful Playgrounds Program, or a similar playground based intervention, have shown changing playgrounds in primary schools to be more conducive to play, and supporting school teachers and students to make use of these spaces can significantly increase levels of physical activity, enjoyment of physical activity and improved social interaction skills (i.e. conflict resolution, and sharing) [17–19]. Peaceful Playgrounds will run a multi-component intervention that incorporates elements of environmental, and social intervention, as well as a teacher professional development to increase physical activity participation.

The environmental component of intervention involves the instillation of multi-coloured playground markings that act as visual prompts for students and teachers to be physically active. The markings will be designed specifically for each participating school, and teaching resources will be provided including games, activity suggestions and units of work that can be performed in relation to the markings. Accompanying equipment and play resources will be provided to schools to support engagement with the playground markings and increase physical activity.

Professional learning support will be offered to teachers at participating schools, which will include strategies for initiating play during breaks, games and activities that support the development of social skills in students such as sharing and self-regulation, and methods of supporting conflict resolution for the playground. The teacher training will also include an online network to provide a platform for teacher self-reflection, discussion forums and opportunities to interact with other participating teachers and the research team.

A Student Ambassador program will be implemented during the intervention to increase the social support students receive to be physically active. Selected students from Stage 3 (years 5 and 6) classes at each school will undertake a student leadership program to develop knowledge and skills to become physical activity ambassadors during break times. The students will be taught techniques from the Peaceful Playgrounds Program to initiate games and encourage social inclusion in their peers.

The Peaceful Playgrounds intervention is expected to increase vigorous physical activity levels in children, encourage participation and enjoyment of physical activity, and structure play in a way that supports socialisation and emotional well-being in children. The HAPPY Study will determine if Peaceful Playgrounds is an effective, efficacious, and scalable intervention.

### Outcomes

The evaluation of the HAPPY Study will be done using three different quantitative instruments to report on the physical activity levels, students’ behaviour in class, and sources of social support to be physically active. Measurement of these three data sets, will allow the research team to analyse the effects of each component of the intervention; how playground markings can effect physical activity levels during lunch and recess; how this increase, as well as social skills taught through the Peaceful Playgrounds intervention may impact students behaviour during class; and how teacher and student development influences from where, and how frequently students’ receive encouragement to by physically active.

### Physical activity during recess and lunch

Observational data will be collected on student’s physical activity levels during lunch and recess breaks, and their utilisation of playground spaces for physical activity recorded using the System for Observing Play and Recreation in Communities (SOPARC) tool (CIAFEL, Portugal: http://activelivingresearch.org/soparc-system-observing-play-and-recreation-communities). This tool will collect student’s sex, their level of physical activity and environmental factors (i.e. time of day, temperature, recess/lunch break, presence of supervision).

SOPARC is a sampling technique in which systematic and periodic scans of individuals and environmental factors within predetermined target areas are made. Each school will have two predetermined target areas, identified by the Principal of participating schools as areas that students use frequently during recess and lunch. Following SOPARC protocols developed in a previous study [20, 21], trained researchers will complete two scans of each areas at both recess and lunch, three times during each term the study is running. The researchers are blind to the conditions of the intervention. Each school in the study will therefore be scanned 12 times each term, for a total of 288 scans across all schools during the study.

A scan involves panning from left to right, and coding each student in the area by sex (male or female), intensity of activity (Sedentary, Walking or Vigorous), and whether they are a Child, Teen, Adult or Senior. Additional data recorded for each scan will include; 1. Weather variables including temperature, rain and UV levels; 2. Time of day the observations were collected, and whether this was a lunch or recess period; 3. What type of activity the students are engaged in (i.e. handball, football, dancing, clapping games) and; 4. Teacher supervision and direction of physical activity. The SOPARC tool has been used by this research team to collect physical activity and sun-safe behaviours in primary schools, and was found to be efficient to use and had high degree of reliably, with an Intraclass Corelation Coefficient (ICC) of .912 [21].

Analysis of data collected with SOPARC will be done at an individual school level, between all schools during the same collection period and then completed on all data collected during the study.

### Behaviour

Student’s on-task behaviour during class time will be measured using the Behavioral Observation of Students in Schools (BOSS) app [22]. Interobserver agreement for the BOSS tool have been consistently reported as high, with kappas ranging from .93 to .98 [23, 24].

Trained researchers will be observing Stage 3 (years 5 and 6) classes following lunch breaks to record the behaviour of students using the BOSS app. Five students will be identified by each participating school, and these students’ (*n* = 30) behaviour will be recorded at each data collection point of the study.

Student behaviour is recorded directly into an iPad using the BOSS app [22], which first codes a target student as engaged (Momentary), or non-engaged (Partial). If a student is identified as being engaged, they are then coded as being either 1. Actively Engaged (i.e. writing, reading aloud, raising a hand, talking to a teacher about the material); 2. Passively Engaged (i.e. listening to a lecture, looking at an academic worksheet, silently reading assigned material). The coding for non-engaged students includes 1. Off-Task Motor (i.e. students being out of their seat, aimlessly flipping pages or worksheets, drawing or writing not related to academic material); 2. Off-Task verbal (i.e. talking to another student about non-academic material, making sounds such as humming, calling out answers or comments to teachers) and; 3. Off-Task Passive (i.e. staring out the window, listening to other students talk about non-academic material, looking around the room).

### Social Support

The Physical Activity Social Support Scale [25] will be given to all students in Stage 3 (years 5 and 6) classes as a survey, and used to collect self-report data measuring sources of adult and peer support of physical activity. Test-retest reliability of the scale is strong (ICC = .88) and the Internal consistency of the items was evaluated at α = .77 [26]. The tool is a 15 min survey with nine questions about how often students receive encouragement to be physically active and from who. The response to each question is divided into adult male, adult female and other children (peers), and anchored to a six-point Likert scale (None = 0, Often = 1, Sometimes =2, Almost Daily = 3, Daily =4,Don’t know = 6). This tool will be used to measure the effects of the teacher training, and student ambassador components of the Peaceful Playgrounds intervention.

### Statistical methods

Using the Statistical Package for Social Sciences (SPSS, v21), descriptive statistics and measures of central tendency will be used to describe the effect of the intervention from baseline to follow-up data in each step of the study.

Regression analysis will be completed for this study, to determine genuine relationships between the intervention, and the outcomes for physical activity levels, students’ on-task behaviour time, and frequency of social support for physical activity. Mixed-model ANCOVAs in SPSS will be computed, and each mixed model will be fitted to the data using the restricted maximum likelihood method. Percentage of occurrence (i.e. frequency) data will be analysed using ×2. For each mixed model, sex will be included as fixed effects, with the school included as a random effect to account for the effects of stepping by school. This is a standard statistical procedure for analysis of clustered datasets [27].

Intra-cluster comparisons will be completed between control and intervention periods, and for analyses involving school level characteristics of physical activity, the unit of analysis will be one scan of one area.

If schools decide to stop participating in the study, data pertaining to that school will not be included in the final analyses. Likewise, if a student selected for the behavioural observation component of the study decides to stop participating in the study their data will not be used in the final analyses. For all other components of the study (i.e., physical activity observation and Social Support), intention to treat principles will be followed and all possible data collected will be analysed.

## Discussion

Physical activity is generally promoted for its well-evidenced positive impact on children’s physical and mental health [28]. However, increased participation in physical activity is also thought to lead to greater of cognitive functioning (information processing), memory, concentration, behaviour and academic achievement for children [29]. The HAPPY Study aims to examine the relationship between physical activity and behavioural well-being in primary school students, and to understand the environmental and societal factors that encourage physical activity in primary school students. This study will measure the Peaceful Playgrounds intervention; whether playground markings increases physical activity levels; if teacher training and a student leadership program encourage students to be more physically active; and whether an increase in physical activity, and social skill development will change students’ behaviour.

The HAPPY study will add to the literature around physical activity in primary schools, the effect physical activity has on behaviour, and whether interventions such as Peaceful Playgrounds successfully impact physical activity and behaviour. Within the physical activity area, the HAPPY Study will provide observational data on physical activity levels in primary school students during recess and lunch breaks. The Peaceful Playgrounds intervention will also provide teachers with resources and training to deliver physical activity sessions outside of the curriculum, so this study will also show the effectiveness of this at increasing physical activity, and encouraging student to be active. While, there is already a large number of studies around effectiveness of playground interventions [13, 14], the Peaceful Playgrounds intervention used in this study is the first we are aware of that combines environmental modifications, teacher development and student leaderships components to influence physical activity levels.

The study will also add to the body of evidence that reports on the effect play (physical activity) can have on children’s socialisation, their creativity, and their behaviour. Currently, there is limited Australian data available on this, and international studies have a focus on academic achievements, rather than the behaviour and on-task time this study will be reporting on.

A key strength of this study is the stepped wedge cluster controlled trial design. The design of the study means that time can be used as a variable to measure the effect of the intervention overtime. The design of the study also enables the research team to run the intervention in all schools over the 12-month study period. While this is thought to be ethical [30], it is also likely to decrease attrition.

If this study is successful in increasing students’ physical activity levels and modifying students’ behaviour, future research could include examining which aspects of the intervention produce which effects.

## Abbreviations

ACTRN: Australian Clinical Trials Registry Number
ANCOVA: Analysis of covariance
BOSS: Behavioral Observation of Students in Schools
CT: Controlled Trial
HAPPY: Healthy Active Peaceful Playgrounds for Youth
HREA: Human Research Ethics Application
ICC: Intra-cluster correlation
MVPA: Moderate Vigorous Physical Activity
NSW: New South Wales
NSWDEC: New South Wales Department of Education and Communities
SERAP: State Education Research Applications Process
SOPARC: System for Observing Play and Recreation in Communities
SPSS: Statistical Package for the Social Sciences
